# Interferon-induced transmembrane protein 3 in the hippocampus: a potential novel target for the therapeutic effects of recombinant human brain natriuretic peptide on sepsis-associated encephalopathy

**DOI:** 10.3389/fnmol.2023.1182005

**Published:** 2023-08-01

**Authors:** Nan Li, Rui-Hang Ma, Er-Fei Zhang, Feng Ge, De-Yu Fang, Jing Zhang, Yan-Ning Zhang, Yan Gao, Li-Chao Hou, Hong-Xu Jin

**Affiliations:** ^1^Department of Emergency Medicine, General Hospital of Northern Theater Command, Shenyang, Liaoning, China; ^2^Department of Anesthesiology and Critical Care Medicine, Xijing Hospital, The Fourth Military Medical University, Xi'an, Shaanxi, China; ^3^Department of Anesthesiology, The Affiliated Hospital of Yan’an University, Yan’an, Shaanxi, China; ^4^Department of Chemistry, Liaoning University of Traditional Chinese Medicine, Shenyang, Liaoning, China; ^5^Department of Intensive Care Unit, Yue Bei People’s Hospital, The Affiliated Hospital of Shantou University, Shaoguan, Guangdong, China; ^6^Department of Nephrology, General Hospital of Northern Theater Command, Shenyang, China; ^7^Department of Anesthesiology, Xiang’an Hospital of Xiamen University, Xiamen, Fujian, China

**Keywords:** sepsis-associated encephalopathy (SAE), recombinant human brain natriuretic peptide (rhBNP), interferon-induced transmembrane protein 3 (IFITM3), inflammation, apoptosis

## Abstract

**Objective:**

This study aims to explore whether interferon-induced transmembrane protein 3 (IFITM3) is involved in recombinant human brain natriuretic peptide (rhBNP)-mediated effects on sepsis-induced cognitive dysfunction in mice.

**Methods:**

The cellular localization and expression level of IFITM3 in the hippocampus were detected. The IFITM3 overexpression was achieved using an intracranial stereotactic system to inject an adeno-associated virus into the hippocampal CA1 region of mice. Field experiments, an elevated plus maze, and conditioned fear memory tests assessed the cognitive impairment in rhBNP-treated septic mice. Finally, in the hippocampus of septic mice, terminal deoxynucleotidyl transferase biotin-dUTP nick end labeling (TUNEL) staining and Immunoblot were used to detect changes in the protein expression of cleaved Caspase-8 and cleaved Caspase-3 in apoptosis-related pathways, and toll-like receptor 4 (TLR4) and nuclear factor κB (NF-κB) p65 in inflammatory pathways.

**Results:**

Fourteen days after cecal ligation and puncture (CLP) surgery, IFITM3 localized in the plasma membrane and cytoplasm of the astrocytes in the hippocampus of septic mice, partially attached to the perivascular and neuronal surfaces, but not expressed in the microglia. The expression of IFITM3 was increased in the astrocytes and neurons in the hippocampus of septic mice, which was selectively inhibited by the administration of rhBNP. Overexpression of IFITM3 resulted in elevated anxiety levels and long-term learning and memory dysfunction, completely abolished the therapeutic effect of rhBNP on cognitive impairment in septic mice, and induced an increase in the number of neuronal apoptosis in the hippocampal CA1 region. The expression levels of cleaved Caspase-3 and cleaved Caspase-8 proteins were significantly increased in the hippocampus, but the expression levels of TLR4 and NF-κB p65 were not increased.

**Conclusion:**

The activation of IFITM3 may be a potential new target for treating sepsis-associated encephalopathy (SAE), and it may be one of the key anti-apoptotic mechanisms in rhBNP exerting its therapeutic effect, providing new insight into the clinical treatment of SAE patients.

## 1. Introduction

Sepsis-associated encephalopathy (SAE) is characterized by diffuse brain dysfunction including inattention, delirium and even coma, that is caused by systemic inflammatory response in the absence of clinical or laboratory evidence of direct CNS infection (Mazeraud et al., 2018). Studies have shown that 8–70% of patients surviving sepsis suffer from decreased living ability, cognitive impairment related to learning and memory, and mental health disorders, resulting in permanent brain dysfunction and reduced health-related quality of life, and it has caused serious socioeconomic problems, increasing social and family burdens (Iwashyna et al., 2010; Widmann and Heneka, 2014). To date, no breakthrough has been made in SAE treatment, and the underlying cause is that the pathogenesis of sepsis/SAE is not fully understood. In our previous study, we found that recombinant human brain natriuretic peptide (rhBNP) can improve the 14-day survival rate of septic mice, possibly by regulating the toll-like receptor 4 (TLR4) and nuclear factor κ B (NF-κB) signaling pathways, reducing the blood–brain barrier (BBB) permeability, inhibiting neuronal apoptosis, and reducing the level of inflammatory cytokines to alleviate pathological brain damage and improve cognitive dysfunction (Li et al., 2020). However, more in-depth molecular mechanisms need further study.

The intervention of the cell signaling pathways may be a new direction for SAE treatment. Interferon-induced transmembrane protein 3 (IFITM3) is a transmembrane protein induced by type I interferon (IFN) acting on the IFN-responsive gene cyclic GMP (cGMP) and has broad-spectrum antiviral activity (McNab et al., 2015). However, its role in other immune-related diseases, such as bacterial infections, is unclear, and the mechanism may be more complex. Studies have shown that IFITM3 is involved in the inflammatory response of the central nervous system and plays a vital role in the pathogenesis of neurodevelopmental diseases related to immune activation, such as schizophrenia, Alzheimer disease, maternal infection virus and disrupting the normal development of the neonatal mouse brain (Ibi et al., 2009; Horvath and Mirnics, 2014; Hur et al., 2020). However, little is known about whether IFITM3 is involved in the occurrence and biological expression of sepsis-induced brain injury and cognitive impairment. By acting on chorionic gonadotropin (CG) receptors, rhBNP regulates the concentration of intracellular cGMP and exerts a series of pharmacological effects. cGMP is an essential effector gene for inducing IFITM3 (Sun et al., 2013). Therefore, we speculate that rhBNP may regulate the IFITM3 signaling pathway for SAE to produce a therapeutic effect. Therefore, this study aimed to observe the localization and expression level of IFITM3 in the brain tissue of the cecal ligation and puncture (CLP)-induced septic mouse model and to preliminarily explore the mechanism of IFITM3 in rhBNP treatment of SAE.

## 2. Materials and methods

### 2.1. Animals and treatment

The animals used in this experiment were adult male C57BL/6 J mice, healthy specific pathogen-free experimental animals, provided by Beijing Wei Tong Li Hua Experimental Animal Technology Co., Ltd., animal permit number: SCXK(Jing) 2018–0002. The experimental study was started 1 week after the animals were acclimated to the environment. This experimental animal breeding standard conforms to the procedures of the Laboratory Animal Center of the Fourth Military Medical University, abides by the requirements of animal ethics, and performs minimal damage to animals through traumatic experimental operations as the basic criteria. Institutional Animal Care and Use Committee (IACUC)” number is IACUC-20210260. The murine CLP sepsis model refers to our previous experimental method (Li et al., 2020). The detailed method is included in the Supplementary Material.

### 2.2. Experimental design

#### 2.2.1. Experiment 1: to observe the effect of rhBNP treatment on the expression of IFITM3 in the hippocampus of septic mice

This experiment used forty-two adult male C57BL/6 J mice, 8–10 weeks old, weighing 20–24 g. They were weighed, numbered, and randomly divided into four groups. The C57BL/6 J mice were randomly divided into the sham operation + vehicle (sham+veh), sham operation + rhBNP 50 μg/kg subcutaneous injection (sham+rhBNP), sepsis + vehicle (CLP + veh), CLP + rhBNP 50 μg/kg groups (CLP + rhBNP). Brain tissues of surviving animals were taken to test IFITM3 expression, with six animals in each group.

#### 2.2.2. Experiment 2: the effects of the overexpression of IFITM3 after rhBNP treatment of sepsis

Thirty male C57BL/6 J mice, 6 weeks old, weighing 15–18 g, were used in this experiment. They were weighed, numbered, and randomly divided into two groups (fifteen in each group): the rAAV-EF1a-mCherry and rAAV-EF1a-IFITM3-P2A-mCherry groups. The two groups of animals were intracranial stereotaxic injected with rAAV-EF1a-mCherry or rAAV-EF1a-IFITM3-P2A-mCherry in the bilateral hippocampal CA1 region. Based on this, the CLP-induced septic mice were treated with rhBNP for 14 days, and the changes in cognitive function were evaluated by the open field test (OFT), elevated plus maze (EPM), and context-related fear test (ten mice in each group). All animals were subjected to behavioral evaluations that survived after the bilateral hippocampal CA1 region injection of rAAV and CLP surgery. After the behavioral tests, the animals were sacrificed. Their brain tissue were taken for Immunoblot to detect the changes in the expression levels of the inflammation-related proteins TLR4 and NF-κB p65 and apoptosis-related proteins cleaved Caspase-8 and cleaved Caspase-3 (four animals in each group). The changes in the apoptosis level of the nerve cells were observed by the TUNEL staining method (three animals in each group).

### 2.3. Administration

#### 2.3.1. Experiment 1

rhBNP was purchased from Nuodikang Biological Pharmaceutical Company, Ltd. (Chengdu, China). According to our previous study results, 50 μg/kg of rhBNP was administered for drug treatment. All animals were intraperitoneally injected with 75 mg/kg of ertapenem once a day, 6 h after surgery, until 3 days after surgery. The animals in the sham+veh and CLP + veh groups were given the same amount of saline 6 h after the operation, and the animals in the sham+rhBNP and CLP + rhBNP groups were given 50 μg/kg of rhBNP (once a day) 6 h after the operation (subcutaneous injection) for 14 consecutive days.

#### 2.3.2. Experiments 2 and 3

Four weeks before the CLP surgery, the animals were stereotaxically injected with adeno-associated rAAV-EF1a-mCherry or rAAV-EF1a-IFITM3-P2A-mCherry virus (BrainVTA Technology Co., Ltd., China) in the CA1 region of the bilateral hippocampus. All animals were intraperitoneally injected with 75 mg/kg of ertapenem once a day, 6 h after CLP surgery until 3 days after surgery. At the same time, 50 μg/kg of rhBNP (once a day, subcutaneous injection) was administered for 14 consecutive days.

### 2.4. Stereotaxic technique of brain and microinjection

After the experimental mice were anesthetized by isoflurane inhalation, they were placed on a digital stereotaxic instrument (Stoelting Co., United States), the heads of the mice were fixed, and the balance was adjusted using anterior tooth adapters and ear bars on both sides. Their skin was cut, and their skulls were exposed. The mouse hippocampal CA1 area coordinates were AP-1.9 mm, ML ± 1.4 mm, DV-1.3 mm, and the hole punch was lightly drilled. After redetermining the position, a microinjection system (Stoelting Co., United States) was used to control a glass electrode microinjector to slowly inject rAAV-EF1a-IFITM3-P2A-mCherry or rAAV-EF1a-mCherry into the bilateral hippocampal CA1 area. The total volume was 300 nL, and the speed was 23 nL/min for about 15 min. After the microinjection stopped, there was a 10 min wait to allow the virus to spread evenly to the tissue. After the needle was slowly pulled out, the head skin was sutured, the surgical area was smeared with aneriodine after surgery, 0.1 mL of 4% ropivacaine was injected locally into the incision, and lidocaine ointment was applied. After the mice were awake for 1 h, they were put back into their cage and were free to move around and eat and drink water.

### 2.5. Behavioral testing

#### 2.5.1. Open field test and elevated plus maze

The OFT is a classic behavioral experiment used to evaluate the animals’ spontaneous activity, anxiety state, and exploratory behavior.

The EPM is based on the animal’s spontaneous fear-like reflex, which is an unconditioned reflex model and an internationally recognized method for measuring anxiety responses.

OFT and EPM were used to detect behavioral changes as previously described (Li et al., 2020). The detailed method is included in the Supplementary Material.

#### 2.5.2. Contextual phased memory

Conditioned fear memory tests can reflect hippocampal-related long-term learning and memory capabilities, so we used this behavioral paradigm to observe context-related differences in fear memory capabilities in mice treated with different methods.

1) On the first day, the mice were placed in the fear box and allowed to move freely for 10 min. After each animal was tested, the fear box was cleaned and wiped repeatedly with 70% alcohol to remove residual odor.
2) On the second day, the mice were gently placed in the fear box for 2 min, and their movement status was observed as the basal value of freezing. Freezing is considered as a defensive posture maintained over 2 s in which the mouse’s body and head do not engage in any movement except for breathing. In the absence of related stimuli, such as sound, light, smell, etc., five consecutive unconditioned stimuli were given at 2 min and 10 s intervals of the experiment (stimulus content: electric shock: 0.7 mA, 2 s; interval: 35–60 s). After each animal was tested, the fear box was cleaned and wiped repeatedly with 70% alcohol to remove residual odor.

On the third day, after 24 h of unconditioned fear training, the mice were gently placed in the same fear box again, and the percentage of freezing time in the total experimental time was recorded when the mice were tested. After each animal was tested, the fear box was cleaned and wiped repeatedly with 70% alcohol to remove residual odor.

### 2.6. Immunohistochemistry

The prepared tissue sections were briefly rinsed in 0.1 M phosphate-buffered saline (PBS) and incubated with 0.1% Triton X-100 for 30 min. Subsequently, the sections were blocked with 0.1 M PBS containing 0.03% Triton X-100 and 5% goat serum for 1 h. The primary antibodies were anti-IFITM3 (Abcam, ab15592, 1:100), anti-glial fibrillary acidic protein (GFAP) (Abcam, ab4648, 1:200), anti-neuronal nuclear protein (NeuN) (Abcam, ab104224, 1:500), and ionized calcium-binding adapter molecule 1 (Iba1) (Novus Biologicals, NB-1028ss, 1:100). The tissue sections were incubated for 2 h at room temperature of 24°C, kept overnight at 4°C, and washed three times with PBS for 5 min. They were incubated in the dark with Alexa Fluor 488-conjugated goat anti-rabbit (Abcam, 1:300) or Alexa Fluor 594-conjugated goat anti-mouse (Abcam, 1:300) secondary antibody dilutions for 2 h at room temperature, respectively. They were washed three times with PBS for 5 min. The slides were mounted in glycerol, observed under a fluorescence microscope, and photographed.

### 2.7. Immunoblot

Protein was extracted from the hippocampus tissues according to standard Invent protocols (Invent, China) as in our previous study (Li et al., 2020). The total, nuclear, and total member proteins from the hippocampus tissues were separated by sodium dodecyl sulfate–polyacrylamide gel electrophoresis (SDS–PAGE), blotted and probed with the following antibodies: anti-IFITM3 (Abcam, ab15592, 1:1,000), anti-TLR4 (ProteinTech, 10,745-1-AP, 1:1,000), anti-NF-κB (ProteinTech, 66,350–1-Ig, 1:1,000), anti-β-actin (ProteinTech, 6,008–1-Ig, 1:2,000), anti-GAPDH (Abcam, ab8245, 1:2,000), anti-Na/K-ATPase (GeneTex, GTX30203), anti-cleaved caspase-8 (Abcam, ab25901, 1:1,000), and anti-cleaved caspase-3 (Abcam, ab13847, 1:1,000). Bradford assays (Bio-Rad Laboratories, Hercules, CA, United States) were used to quantify the protein concentrations. The blots were visualized with a chemiluminescence system (Amersham Bioscience, Buckinghamshire, United Kingdom), and the signals were quantified by densitometry. ImageJ was used to measured densities of blots, and the relative density was the ratio of target protein to GAPDH.

### 2.8. Terminal deoxynucleotidyl transferase biotin-dUTP nick end labeling staining

A commercial terminal deoxynucleotidyl transferase biotin-dUTP nick end labeling (TUNEL) kit was used according to the manufacturer’s instructions to detect changes in neuronal apoptosis (Roche Ltd., 11,684,795,910).

### 2.9. Statistical methods

Analysis of the data of the OFT and EPM experiments: the average movement speed, the number of grid crossings, and the activity time of the animals in the central area were observed using the OFT. The anxiety state of the animals was evaluated using the EPM experiment, entering the open arm time percentage (% time ratio in the open arm) = (movement time in the open arm / total experimental time) × 100%.

Data analysis of the fear memory behavior: freezing time ratio of the experimental animals during training and testing (% freezing time ratio) = (freezing time / total experimental time) × 100%.

GraphPad Prism 6.0 software was used for statistical analysis. The mean ± standard error ( $\bar{x}$ ± SE) was used to represent the measurement data. Measurement data with normal distribution were analyzed using the *t*-test and a one-way ANOVA test followed by Tukey’s test for multiple comparisons. Kruskal-Wallis test was for data with abnormal distribution. All statistical tests were two-tailed probability tests. *p* < 0.05 indicated statistical significance.

## 3. Results

### 3.1. Recombinant human brain natriuretic peptide regulates the expression of IFITM3 in the hippocampal astrocytes and neurons of septic mice

**Figure 1:**
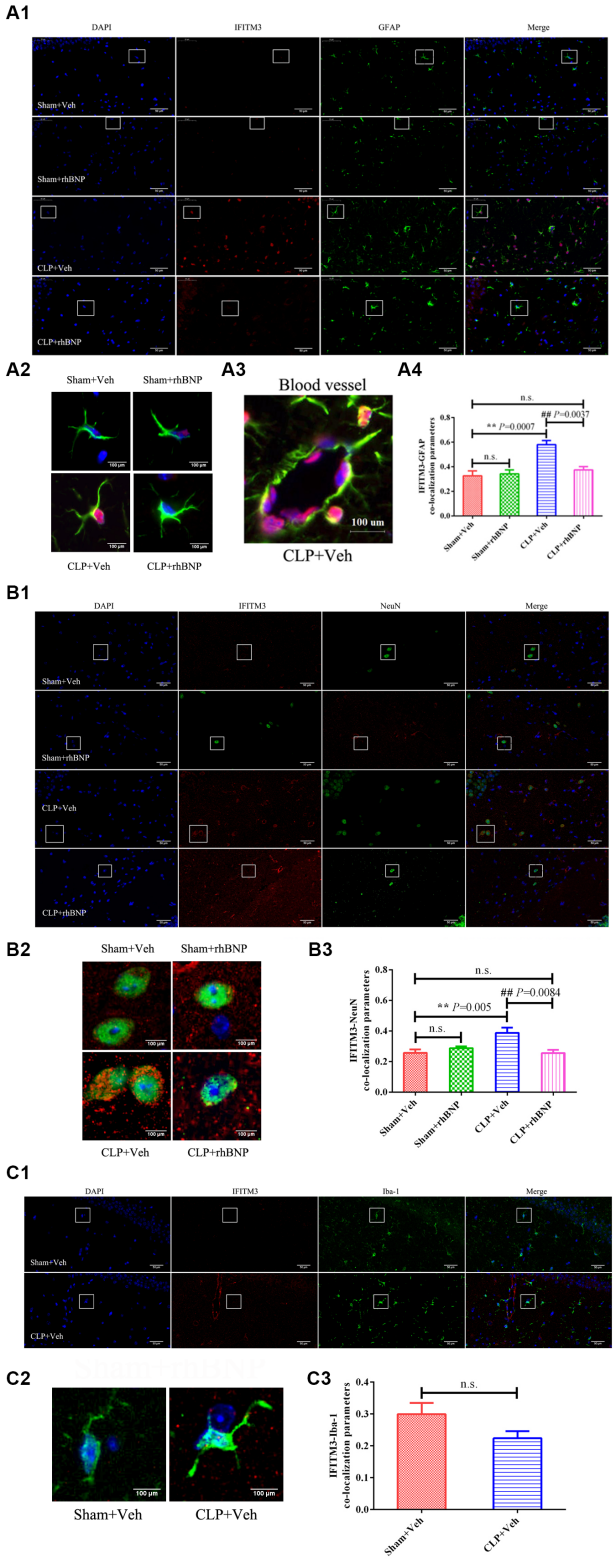
Recombinant human brain natriuretic peptide (rhBNP) treatment selectively inhibited astroglial and neuronal IFITM3 activation following sepsis-associated encephalopathy (SAE). **(A)** Hippocampal IFITM3 protein levels after cecal ligation and puncture (CLP) operation with or without rhBNP treatment. Double immunostaining for IFITM3 [red in **(A-C)**], glial fibrillary acidic protein (GFAP, green in the third panels: A1, A2, and A3), neuronal nuclear protein (NeuN, green in the third panels: B1 and B2), and ionized calcium-binding adapter molecule 1 (Iba1, green in the third panels: C1 and C2), 4,6-diamino-2-phenyl indole [DAPI, blue in **(A-C)**], in the hippocampus after the CLP operation (*n* = 3 in each group). ^*^p < 0.05, ^**^p < 0.01, compared with the sham+veh group, ^#^*p* < 0.05, *^##^p* < 0.01, compared with the CLP + veh group. Scale bar = 100 μm.

As shown in Figure 1, the use of glial fibrillary acidic protein (GFAP, a marker for astrocytes; Figure 1A), neuronal nuclear protein (NeuN, a marker for mature neurons; Figure 1B), or ionized calcium-binding adapter molecule 1 (Iba1 microglia marker; Figure 1C), and double immunostaining for IFITM3 revealed that IFITM3 immunoreactivity was predominantly colocalized with GFAP-positive astrocytes (Figure 1A) and NeuN-positive neurons (Figure 1B), but without Iba1-positive microglia (Figure 1C), suggesting that astrocytes and neurons express IFITM3 after CLP-induced septic mice. At the same time, it could be seen that the end feet of the astrocytes were connected to form blood vessels, which coexpress with IFITM3 (Figure 1A). The semi-quantitative analysis of the colocalization coefficient of IFITM3 and different types of cells showed that compared with the sham + veh group, the expression level of IFITM3 in the hippocampal astrocytes of the CLP + veh group was significantly increased (sham + veh: 0.33 ± 0.04 vs. CLP + veh: 0.58 ± 0.03, *p =* 0.0007), with a statistically significant difference. Compared with the CLP + veh group, the expression level of IFITM3 in the hippocampus of mice in the CLP + rhBNP group was decreased (CLP + veh: 0.58 ± 0.03 vs. CLP + rhBNP: 0.37 ± 0.03, *p =* 0.0037), with significant statistical significance learning differences. There was no difference between the mice in the sham+veh and sham+rhBNP groups (sham + veh: 0.33 ± 0.04 vs. sham + rhBNP: 0.34 ± 0.03, *p* = 0.10), which was not statistically significant (Figure 1A). Compared with the sham + veh group, the expression level of IFITM3 in the hippocampal neurons of the CLP + veh group was increased (sham+veh: 0.26 ± 0.02 vs. CLP + veh: 0.39 ± 0.03, *p =* 0.005), which was statistically significant. Compared with the CLP + veh group, the expression level of IFITM3 in the hippocampal neurons of the CLP + rhBNP group was decreased (CLP + veh: 0.39 ± 0.03 vs. CLP + rhBNP: 0.26 ± 0.04, *p =* 0.0084), with a statistically significant difference. There was no difference between the mice in the sham+veh and sham+rhBNP groups (sham+veh: 0.26 ± 0.02 vs. sham+rhBNP: 0.29 ± 0.01, *p* = 0.48), which was not statistically significant (Figure 1B). Additionally, there was no significant change in IFITM3 in the hippocampal microglia of mice in the CLP + veh group (sham+veh: 0.30 ± 0.04 vs. CLP + veh: 0.22 ± 0.02, *p =* 0.10), which was not statistically significant (Figure 1C).

### 3.2. Recombinant human brain natriuretic peptide can reverse cecal ligation puncture-induced upregulation of IFITM3 expression in the hippocampus of septic mice

**Figure 2:**
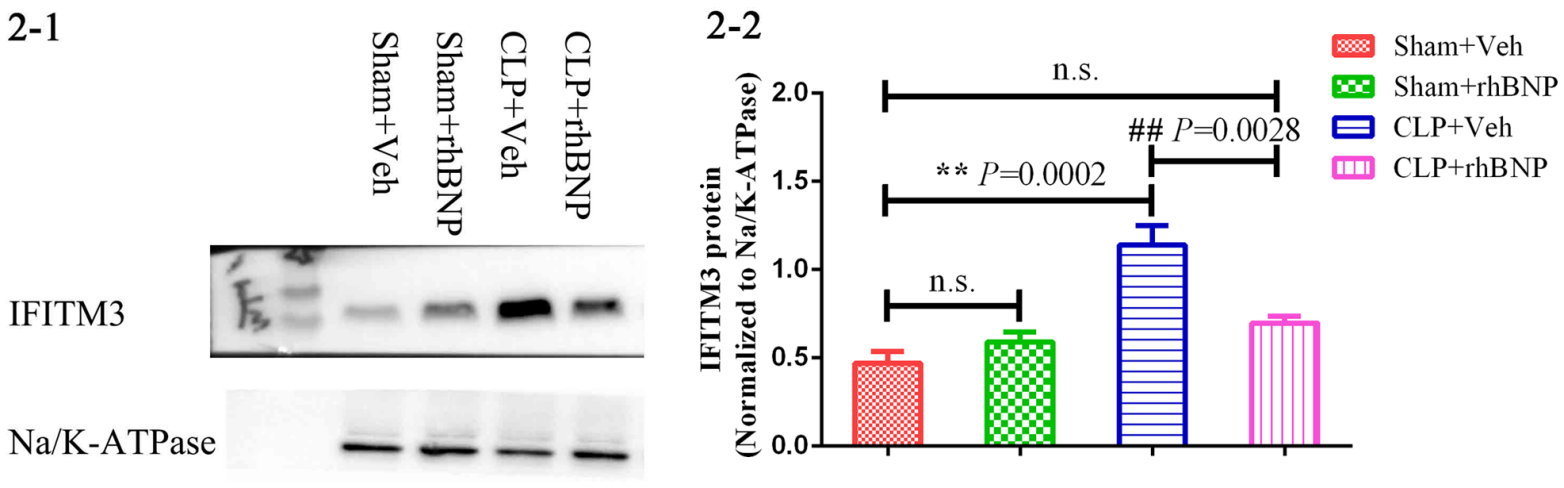
IFITM3 protein levels in the hippocampal CA1 region in CLP-induced septic mice. The brain samples were harvested to measure the levels of IFITM3 protein 14 days after sham or CLP operations. Values are mean ± structural equation modeling ( $\bar{x}$ ± S.E.M.) (*n* = 3 in each group), ^*^p < 0.05, ^**^p < 0.01, versus the sham + veh group, ^#^*p* < 0.05, ^##^*p* < 0.01, versus the CLP + Veh group.

The expression level of IFITM3 in the hippocampus of mice in each group was detected by Immunoblot. The results are shown in Figure 2. The expression of IFITM3 in the hippocampus of the mice in the sham+veh group was very low, and after CLP-induced sepsis, the level of IFITM3 in the hippocampus of the CLP + veh group was significantly increased (sham+veh: 0.47 ± 0.07 vs. CLP + veh: 1.14 ± 0.11, *p =* 0.0002), which was statistically significant. After treatment with rhBNP, the expression level of IFITM3 in the hippocampus of mice in the CLP + rhBNP group decreased (CLP + veh: 1.14 ± 0.11 vs. CLP + rhBNP: 0.70 ± 0.04, *p =* 0.0028), which was statistically significant. Compared with the sham+veh group, there was no significant change in IFITM3 in the hippocampus of the mice in the sham+rhBNP group (sham+veh: 0.47 ± 0.07 vs. sham + rhBNP: 0.59 ± 0.06, *p* = 0.0592), and there was no statistical significance.

### 3.3. The overexpression of IFITM3 by injecting rAAV-EF1a-IFITM3-P2A-mCherry in the hippocampus of septic mice before recombinant human brain natriuretic peptide treatment can completely cancel the therapeutic effect of recombinant human brain natriuretic peptide on anxiety, exploration, learning, and memory dysfunction in septic mice

**Figure 3:**
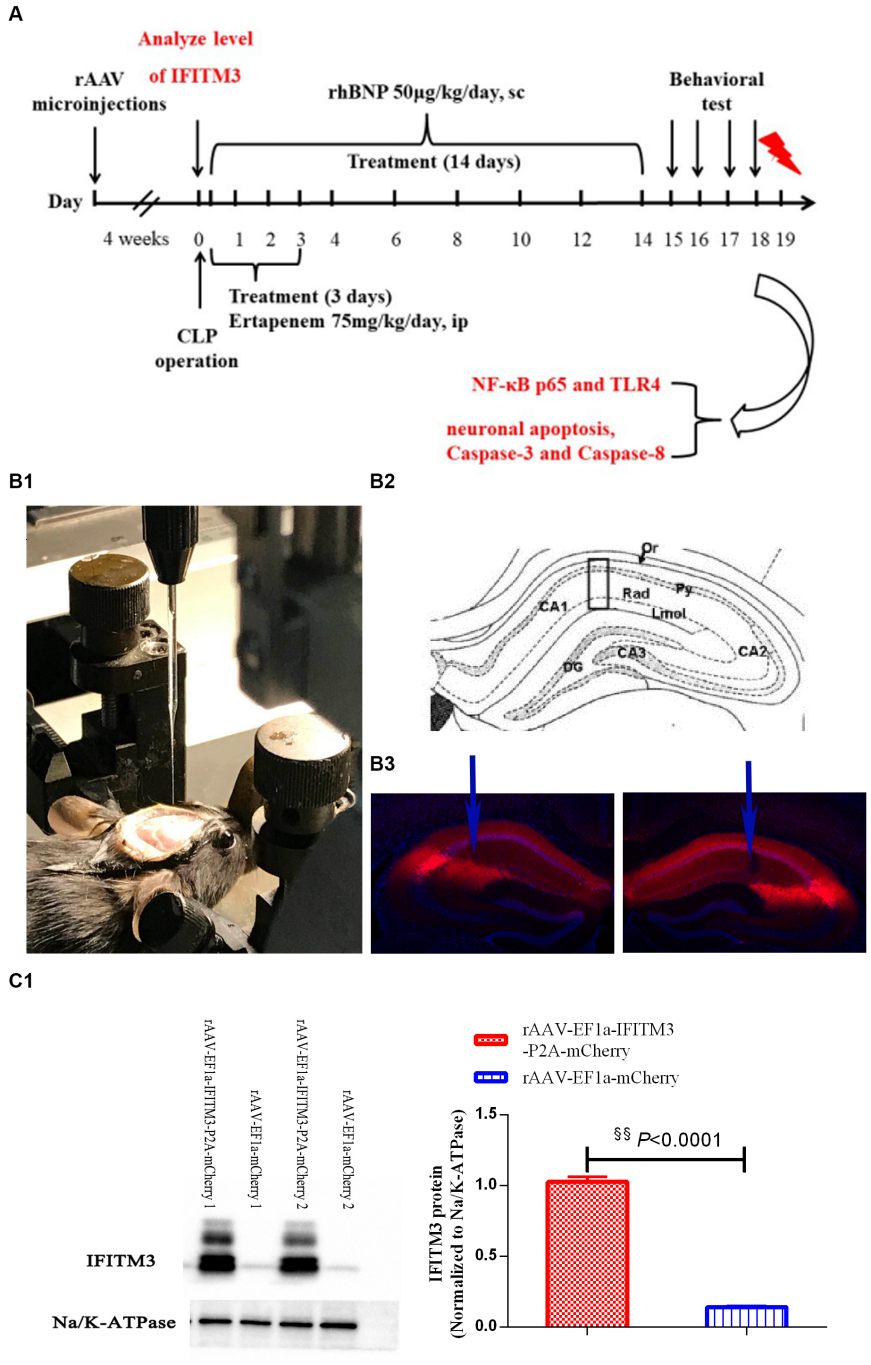
Overexpression of IFITM3 protein and identification of the adeno-associated virus in the hippocampal CA1 region. **(A)** A diagram of the experimental design. **(B)** a diagram of injection of rAAV-EF1a-mCherry or rAAV-EF1a-IFITM3-P2A-mCherry in the hippocampal CA1 region. **(C)** Immunoblot analysis showed that the expression of IFITM3 protein was induced by injection of rAAV-EF1a-mCherry or rAAV-EF1a-IFITM3-P2A-mCherry in the hippocampal CA1 region in two representative animals. Values are means ± structural equation modeling ( $\bar{x}$ ± S.E.M.) (*n* = 4 in each group). ^§§^*p* < 0.01, versus the rAAV-EF1a-mCherry group.

Our previous research showed that rhBNP significantly mitigated cognitive dysfunction and anxiety (Li et al., 2020). In part, we upregulated the expression level of IFITM3 to determine whether it is a key molecule in the effect and mechanism of rhBNP on cognitive dysfunction in septic mice. Due to the lack of IFITM3-selective agonists and antagonists, we microinjected an adeno-associated virus (rAAV-EF1a-IFITM3-P2A-mCherry or rAAV-EF1a-mCherry) containing IFITM3 or an empty vector into the CA1 region of the bilateral mouse hippocampus. Intracranial stereotaxic injection of rAAV-EF1a-IFITM3-P2A-mCherry or rAAV-EF1a-mCherry was given into the CA1 region of the hippocampus of mice (Figure 3A), and 4 weeks later, immunofluorescence staining of the brain tissue was performed. Red fluorescence was brighter in the bilateral CA1 region of hippocampus tissue than other regions (Figure 3B). Immunoblot analysis showed that compared with mice injected with rAAV-EF1a-mCherry in the hippocampal CA1 region, the level of IFITM3 in the hippocampus of mice injected with rAAV-EF1a-IFITM3-P2A-mCherry in the hippocampal CA1 region was overexpressed (Figure 3C, rAAV- EF1a-mCherry vs. rAAV-EF1a-IFITM3-P2A-mCherry groups: 0.14 ± 0.01 vs. 1.03 ± 0.04, *p* < 0.0001), with a statistically significant difference.

**Figure 4:**
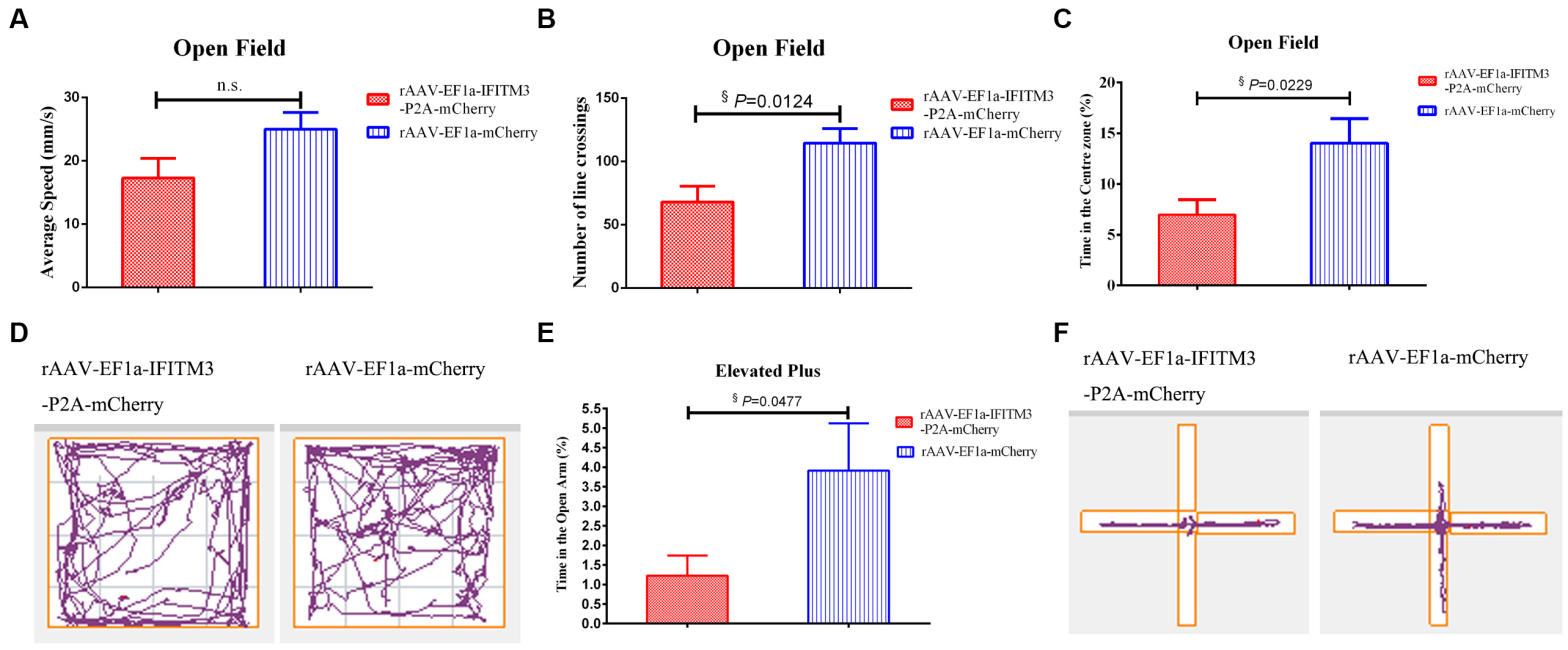
Overexpression of IFITM3 on rhBNP improves anxiety and exploration in mice with sepsis. **(A)** Average speed, **(B)** number of line crossings, **(C)** time in the center zone, **(D)** motion trajectory map in the open field, **(E)** proportion of exploration time in the open arm, and **(F)** motion trajectory in the elevated plus maze. Values are mean ± structural equation modeling ( $\bar{x}$ ± S.E.M.) (*n* = 10 in each group), ^§^*p* < 0.05, versus the rAAV-EF1a-mCherry group.

In the OFT experiment, compared with septic mice injected with rAAV-EF1a-mCherry in the hippocampal CA1 region, the mice in the hippocampal-injected rAAV-EF1a-IFITM3-P2A-mCherry group, the number of crossings exhibited reduced (Figure 4B, rAAV-EF1a-mCherry vs. rAAV-EF1a-IFITM3-P2A-mCherry groups: 114.5 ± 11.57 vs. 68.08 ± 12.33, *p* = 0.0124) and the percentage of time spent in the central zone decreased (Figure 4C, rAAV-EF1a-mCherry vs. rAAV-EF1a-IFITM3-P2A-mCherry groups: 14.04 ± 2.411 vs. 6.991 ± 1.484, *p* = 0.0229). There was a statistical difference in the number of crossings and the time spent in the central zone but no statistical significance in exercise speed between the two groups (Figure 4A, rAAV-EF1a-mCherry vs. rAAV-EF1a-IFITM3-P2A-mCherry groups: 25.00 ± 2.64 mm/s vs. 17.28 ± 3.11 mm/s, *p* = 0.0794). Figure 4D shows the trajectory of one representative mouse in different intervention groups in the OFT. Compared with the septic mice injected with rAAV-EF1a-mCherry in the hippocampal CA1 region, the percentage of active time in the elevated open arm area decreased in the hippocampal-injected rAAV-EF1a-IFITM3-P2A-mCherry group in CLP-induced mice (Figure 4E, rAAV-EF1a-mCherry vs. rAAV-EF1a-IFITM3-P2A-mCherry groups: 3.913 ± 1.206 vs. 1.223 ± 0.5183, *p* = 0.0477), which was statistically significant. Figure 4F shows the trajectory that one representative animal moved in the EPM. It is suggested that overexpression of IFITM3 aggravates the exploration ability of septic mice and increases their anxiety level, which completely abolishes the therapeutic effect of rhBNP.

**Figure 5:**
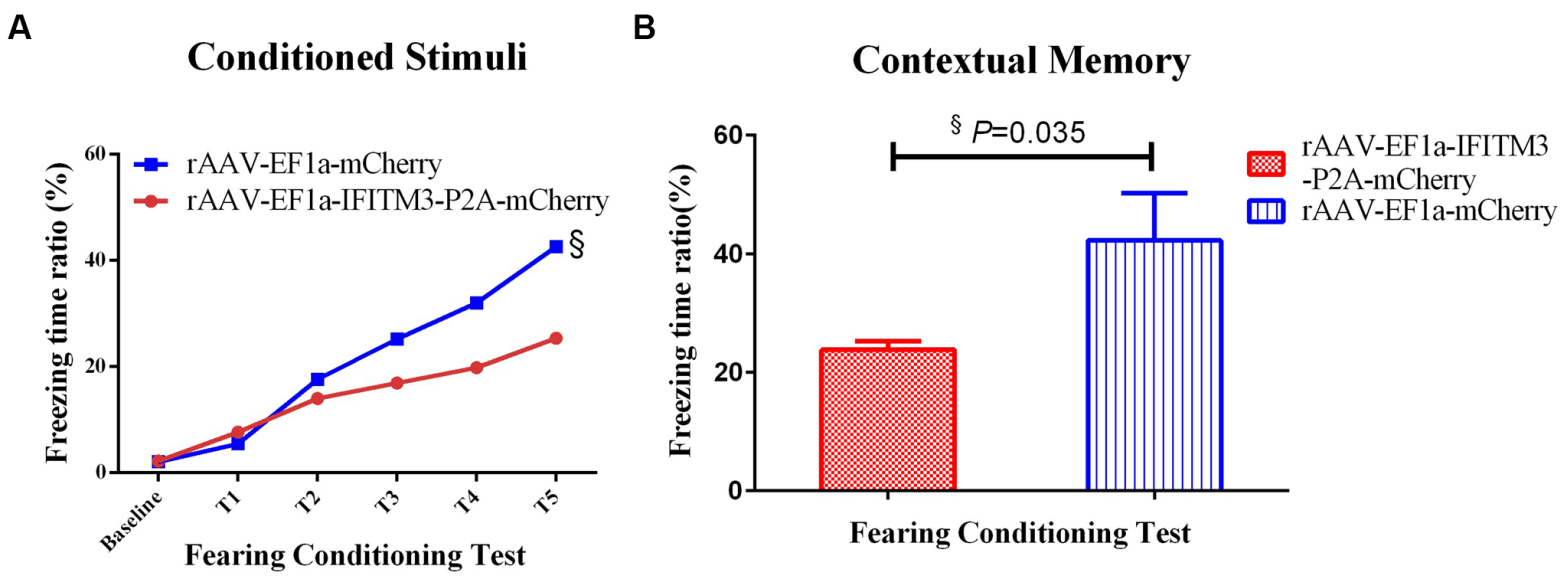
Overexpression of IFITM3 on the effect of rhBNP on improving the contextual fearing memory ability of sepsis mice. **(A)** Freezing time ratio after receiving a foot shock five times (unconditioned stimulation). **(B)** Freezing time ratio in the contextual fear memory test. Values are mean ± structural equation modeling ( $\bar{x}$ ± S.E.M.) (*n* = 10 in each group), ^§^*p* < 0.05, versus the rAAV-EF1a-mCherry group.

In the condition-related fear test, both groups of animals showed freezing behavior after receiving the unconditioned stimulus of foot shock. After subplantar shock, compared with the septic mice injected with rAAV-EF1a-mCherry in the hippocampal CA1 region, the percentage of freezing time in the hippocampus-injected rAAV-EF1a-IFITM3-P2A-mCherry group was statistically significant (Figure 5A). Memory retrieval was performed 24 h after the end of training. The results showed that compared with septic mice injected with rAAV-EF1a-mCherry in the hippocampal CA1 region, the freezing time reduced of the hippocampal-injected rAAV-EF1a-IFITM3-P2A-mCherry group. The percentage of reduction was statistically significant (Figure 5B, rAAV-EF1a-mCherry vs. rAAV-EF1a-IFITM3-P2A-mCherry groups: 42.25 ± 7.961 vs. 23.81 ± 1.405, *p* = 0.035).

### 3.4. Overexpression of IFITM3 could not abolish the inhibitory effect of recombinant human brain natriuretic peptide on the expression levels of toll-like receptor 4 and nuclear factor κB p65 proteins in the hippocampus of septic mice

**Figure 6:**
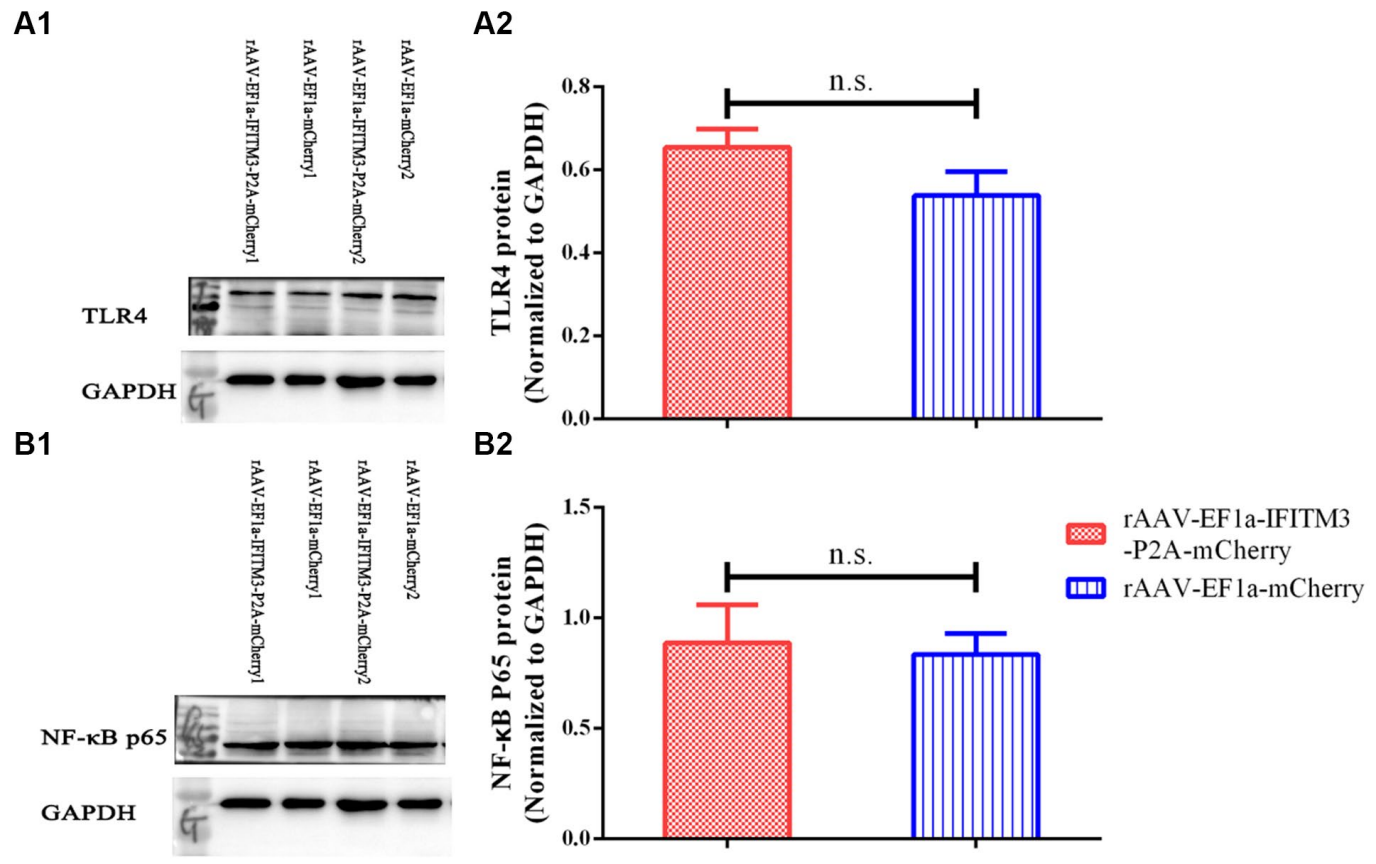
Overexpression of IFITM3 protein did not abolish the inhibitory effect of rhBNP on toll-like receptor 4 (TLR4) and nuclear factor κ B (NF-κB) p65 protein expression levels. **(A)** Level of TLR4 protein, **(B)** level of NF-κB p65 protein in two representative animals. Values are mean ± structural equation modeling ( $\bar{x}$ ± S.E.M.) (*n* = 3 in each group), n.s., versus the rAAV-EF1a-mCherry group.

Our previous research suggested that rhBNP therapy reduced the level of inflammatory cytokines in the hippocampus, possibly *via* inhibiting the TLR4-NF-κB pathway (Li et al., 2020). In this study, we determined whether there were changes in the protein expression of TLR4 and NF-κB p65 in the hippocampus of mice in the rAAV-EF1a-mCherry and rAAV-EF1a-IFITM3-P2A-mCherry groups after the behavioral tests. Compared with the septic mice injected with rAAV-EF1a-mCherry, the expression levels of TLR4 protein (Figure 6A, rAAV-EF1a-mCherry vs. rAAV-EF1a-IFITM3-P2A-mCherry groups: 0.54 ± 0.06 vs. 0.65 ± 0.04, *p* = 0.19) and NF-κB p65 protein (Figure 6B, rAAV-EF1a-mCherry vs. rAAV-EF1a-IFITM3-P2A-mCherry groups: 0.84 ± 0.09 vs. 0.89 ± 0.17, *p* = 0.81) of the animals in the hippocampus injected with rAAV-EF1a-IFITM3-P2A-mCherry were not statistically significant.

### 3.5. Overexpression of IFITM3 abrogates the therapeutic effect of recombinant human brain natriuretic peptide on reducing the number of apoptotic cells and inhibiting the expression levels of cleaved Caspase-8 and cleaved Caspase-3 proteins in the hippocampus of septic mice

**Figure 7:**
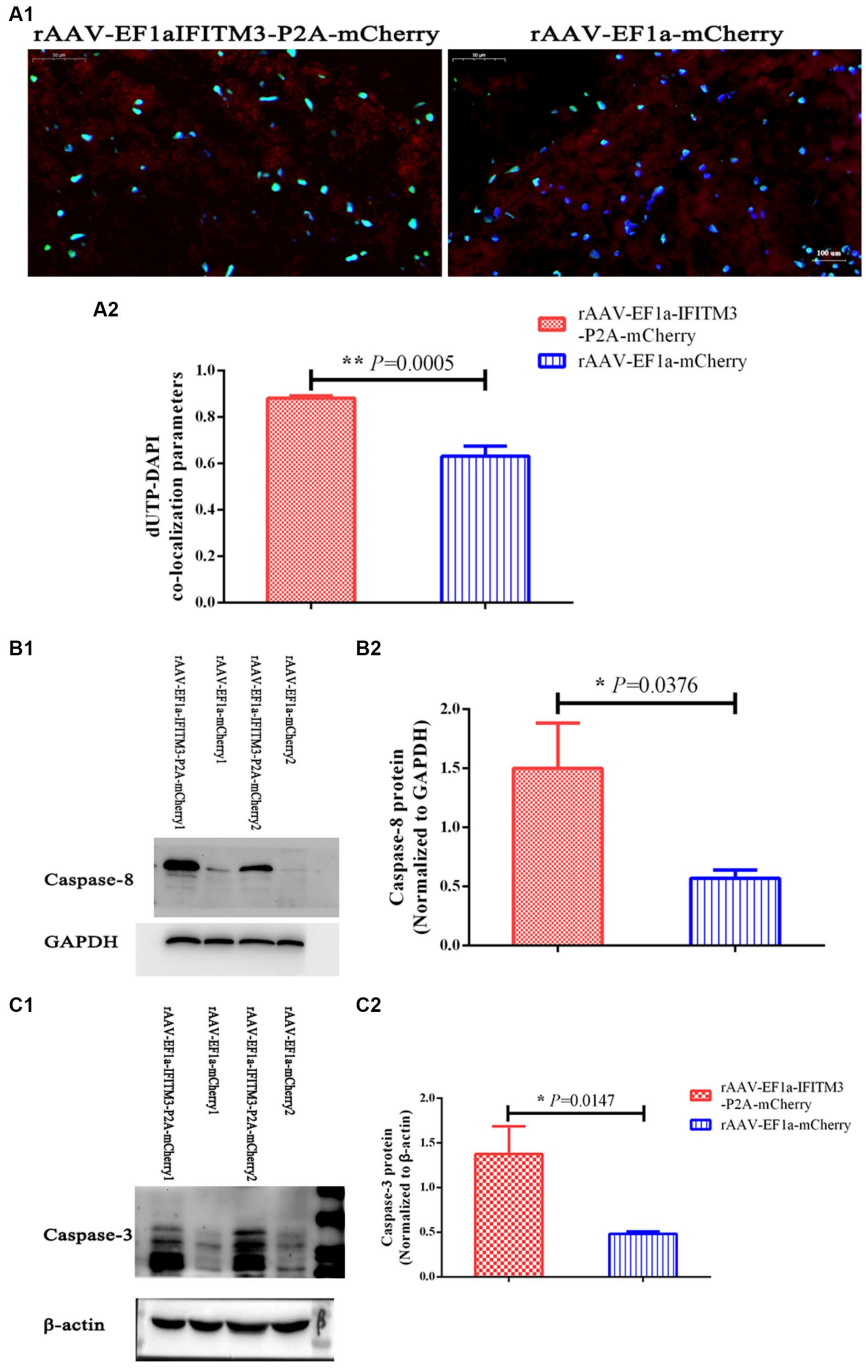
Overexpression of IFITM3 protein abolished the therapeutic effect of rhBNP on reducing the level of apoptosis in the brain tissue of septic mice. **(A)** Terminal deoxynucleotidyl transferase biotin-dUTP nick end labeling (TUNEL) staining. Values are mean ± structural equation modeling ( $\bar{x}$ ± S.E.M.) (*n* = 3 in each group). **(B)** Level of cleaved Caspase-8 protein and **(C)** cleaved Caspase-3 protein in two representative animals. Values are mean ± S.E.M, (*n* = 4 in each group), ^§^*p* < 0.05, ^§§^*p* < 0.01, versus the rAAV-EF1a-mCherry group.

We demonstrated that rhBNP treatment significantly reduced pathological changes in the brain of CLP mice, including the BBB permeability and neuronal apoptosis, as previously described (Li et al., 2020). In the present research, the results of TUNEL staining showed that, compared with the septic mice injected with rAAV-EF1a-mCherry in the hippocampal CA1 region, the number of apoptotic cells in the hippocampus of the animals with rAAV-EF1a-IFITM3-P2A-mCherry increased (Figure 7A, rAAV-EF1a-mCherry vs. rAAV-EF1a-IFITM3-P2A-mCherry groups: 0.63 ± 0.04 vs. 0.88 ± 0.01, *p* = 0.0005), which was statistically significant. Immunoblot results showed that, compared with the septic mice injected with rAAV-EF1a-mCherry, the cleaved Caspase-8 protein in the hippocampus of the animals with rAAV-EF1a-IFITM3-P2A-mCherry (Figure 7B, rAAV-EF1a-mCherry vs. rAAV-EF1a-IFITM3-P2A-mCherry groups: 0.57 ± 0.07 vs. 1.5 ± 0.38, *p* = 0.0376) and cleaved Caspase-3 protein (Figure 7C, rAAV-EF1a-mCherry vs. rAAV-EF1a-IFITM3-P2A-mCherry groups: 0.48 ± 0.02 vs. 1.38 ± 0.31, *p* = 0.0147) expression levels increased, which was statistically significant.

## 4. Discussion

The above results showed that CLP induced overexpression of IFITM3 in the hippocampus of septic mice, mainly on the surface of the astrocyte membrane and cytoplasm and on the surface of neurons, but not on the microglia. Treatment with rhBNP selectively inhibited the activation of IFITM3 in the astrocytes and neurons and decreased its expression level. CLP induces IFITM3 activation in the hippocampus of septic mice, and rhBNP treatment can reverse this effect. Overexpression of IFITM3 abolished the therapeutic effect of rhBNP on the long-term context-related memory ability of septic mice. IFITM3 is not involved in the regulation of the TLR4-NF-κB signaling pathway by rhBNP. Overexpression of IFITM3 abrogated the therapeutic effect of rhBNP on reducing the expression levels of cleaved Caspase-8 and cleaved Caspase-3 and reducing the number of apoptotic cells in hippocampal tissue. It was a key to the target that rhBNP improved brain pathological damage and cognitive dysfunction in septic mice by inhibiting the apoptosis level increase of hippocampal tissue.

Although IFITM3 is increased in the brains of patients with neurological and psychiatric disorders, its role in the central nervous system is largely unknown. Our study found that 14 days after CLP surgery, the expression of IFITM3 in the hippocampal CA1 region of septic mice was increased, it was localized in the astrocyte membrane and cytoplasm, and was positively expressed in the neuron cell membrane, which was different from previous research. Ibi D. et al. showed that after inducing an immune challenge with polyinosinic:polycytidylic acid (polyI:C, synthetic double-stranded RNA) in the early developmental stage, only IFITM3 expression was observed in mouse brain tissue in the astrocyte glial cells, but the positive expression was not found in the neurons and microglia (Ibi et al., 2009). Another study showed that polyI:C treatment activated cultured astrocytes, which could induce an increase in the mRNA levels of inflammatory cytokines and *Ifitm3.* Neurite development was impaired when neurons were cultured with a conditioned medium of polyI:C-treated astrocytes (polyI:C-ACM). An *ifitm3*^−/−^ astrocyte-conditioned medium reverses polyI:C-ACM-induced neurodevelopmental abnormalities. In addition, in polyI:C-treated wild-type mice, microtubule-associated protein 2 (MAP2) expression in the frontal cortex, spinous process density, and dendritic complexity were reduced, and the animals developed significant memory impairments, but not in *ifitm3*^−/−^ mice for neuronal damage and cognitive dysfunction (Ibi et al., 2013). Elisabeth Harmon et al. demonstrated that ischemic injury caused the induction of IFITM3 in aged brains following stroke. Next, they found that LPS and pro-inflammatory cytokines treatment increased Ifitm3 mRNA in primary microglia and sim-A9 cells. However, there were not directed evidences showed that stroke and other inflammatory conditions did not induce astrocyte glial cells and neurons IFITM3 expression in different region of aged brain (Harmon et al., 2022). We speculate that the reasons for these differences may be closely related to different models of neurological diseases, different brain regions, and different age stages of animals. Astrocytes are essential functional cells of the nervous system. After being attacked by the immune system, astrocytes in the hippocampus and different regions are activated, releasing a large number of cytokines and toxins, causing irreversible damage to neurons and long-term damage to animals in learning and memory dysfunction (Ibi et al., 2013). Therefore, we have reason to believe that during the development of sepsis, astrocytes in the central nervous system are overactivated and release IFITM3, which acts on neuronal surface sites and disrupts the glial–neuron interaction, thereby damaging the brain.

Studies have shown that cGMP–adenosine monophosphate (AMP) synthase is a cytoplasmic DNA sensor that induces type I IFN production by producing the second messenger cGMP (Sun et al., 2013). McKenzie et al. have shown that atrial natriuretic peptide (ANP)-stimulated rat forebrain primary astrocytes can increase cGMP levels in a time- and concentration-dependent manner (McKenzie, 1992; Fernandes et al., 2009). Several years later, it was successfully confirmed that B-type natriuretic peptide (BNP) and C-type natriuretic peptide (CNP) could stimulate rodent primary astrocytes, increasing cGMP levels and different levels of accumulation in various brain regions, such as the median preoptic area, olfactory bulb, hippocampus, and medial amygdala. In a cGMP-dependent manner, the natriuretic peptides receptors (NPRs) are involved in neuron–gliacyte communication, cerebral blood flow regulation, and neuroinflammation (Prado et al., 2010). Given the vital role of cGMP in the signaling pathway mediated by IFN and NPs, we speculate that rhBNP may have a certain effect on the expression of IFITM3 in the brain astrocytes of mice with sepsis. Our study observed that many inflammatory cytokines were synthesized in the brain during the development of sepsis, resulting in excessive immune activation of nerve cells in the cerebral cortex and hippocampus. The expression of IFITM3 in the hippocampal astrocytes and neurons increased. Administration of rhBNP could selectively inhibit the expression of IFITM3 in the hippocampal astrocytes of septic mice. At the same time, an rAAV-EF1a-IFITM3-P2A-mCherry virus injection induced the overexpression of IFITM3. The animals showed increased anxiety levels and long-term learning and memory impairment, which completely cancels the therapeutic effect of rhBNP on cognitive impairment in septic mice. It suggests that IFITM3 is a crucial molecule in the role and mechanism of disease-related brain pathological damage and cognitive dysfunction.

So how does overexpression of IFITM3 abrogate the therapeutic effect of rhBNP? Studies have shown that IFITM proteins play essential roles in various biological processes, such as immune cell signaling, germ cell homing and maturation, and bone mineralization, among which IFITM3 plays the most significant role in antiviral activity. However, its role in various immune processes, such as cell adhesion, inflammation, and proliferation, has been poorly studied. Nakajima et al. demonstrated for the first time that lipopolysaccharide (LPS) could induce the upregulation of IFITM3 expression in cultured astrocytes and administration of neutralizing antibodies to IFN-β and tumor necrosis factor α (TNF-α) could completely or partially inhibit the expression of IFITM3 (Nakajima et al., 2014). Pharmacological experiments showed that the administration of TLR4 and NF-κB p65 protein inhibitors could reduce the LPS-induced increase in the expression of IFITM3 in astrocytes and reduce the production of inflammatory cytokines. Porcine IFITM3 overexpression inhibited the LPS-induced inflammatory response in PK15 cells and downregulated the expression of TLR4, NF-κB, and p38 proteins, and TNF-α, suggesting that IFITM3 is involved in the TLR4-NF-κB signaling pathway and plays a vital role in the inflammatory response (Li et al., 2017). The proinflammatory cytokine IL-6 can strongly induce increased expression of IFITM3, and infection of IFITM3-deficient mice with cytomegalovirus resulted in higher levels of IL-6 production (Stacey et al., 2017). Furthermore, IFITM3 regulated IFN-β production induced by Sendai virus infection in human cell lines (Shi et al., 2017). That is, IFITM3 overexpression inhibits cytokine production, whereas its knockdown has the opposite effect. Since IFN-β itself induces the expression of IFITM3, which implies that IFITM3 plays a role in the negative feedback of the type I IFN pathway, this finding needs to be tested experimentally *in vivo* in mouse disease models (Saitou et al., 2002), and inflammatory cytokine production will arouse great interest for researchers and clinicians in the pathogenesis of immune activation-related diseases. However, our study yielded completely different results. Overexpression of IFITM3 did not induce increased expression levels of TLR4 and NF-κB p65 and failed to affect the regulation of the TLR4-NF-κB signaling pathway in the brain of septic mice by rhBNP. IFITM3 is induced by viral infection and cytokines, it is likely that it acts downstream of cytokine activation, which may partly explain why its overexpression does not impact inflammatory activation and signaling mediators TLR4 and NF-κB. This would also be consistent with its feedback participation in inhibiting viral induction of IFN-β. Therefore, we speculate that IFITM3 does not play a key role in rhBNP attenuating the central nervous system inflammatory response and treating cognitive dysfunction in septic mice.

In addition to being involved in immune cell regulation, IFITM proteins are involved in germ cell homing and maturation during embryonic development (Tanaka et al., 2005). Studies have shown that IFITM3 plays a key role in tumor pathogenesis by regulating apoptosis, antiproliferative, and differentiation activities (Rajapaksa et al., 2020). The expression level of *IFITM3* in astrocytoma cells is higher than in normal astrocytes, and the knockdown of *IFITM3* by RNAi successfully inhibited cell proliferation and migration and promoted the apoptosis of glioma cells (Zhao et al., 2013). Tong et al. (2022) reported that MiR-487b suppressed inflammation and neuronal apoptosis in spinal cord injury by targeted Ifitm3. Involvement of IFITM3 in these processes demonstrates its role for keeping immune and nervous system equilibrium. However, whether IFITM3 plays a role in sepsis-induced neuronal apoptosis has not been reported. Using TUNEL staining and Immunoblot, we observed that overexpression of IFITM3 can induce an increase in the number of apoptosis cells in the hippocampal CA1 region and the expression levels of cleaved Caspase-8 and cleaved Caspase-3 proteins in the hippocampus significantly increased in rhBNP-treated sepsis mice. Our results confirmed for the first time that IFITM3 is a pro-apoptotic protein involved in the occurrence of pathological brain damage caused by sepsis. It is one of the key molecules in the role and mechanism of cognitive dysfunction in SAE.

In addition, some studies have reported that IFITM3 mRNA is abundantly expressed in the meninges and blood vessels of the forebrain cortex of patients with schizophrenia, and it is negatively correlated with the level of gamma aminobutyric acid (GABA)-related mRNA. There will also be a considerable lack of GABA-related neurotransmitters, resulting in anxiety, fatigue, and other emotions, as well as learning and memory deficits (Siegel et al., 2014). Our study observed that IFITM3 was expressed in the astrocytes in the CA1 region of the hippocampus of septic mice by the immunofluorescence double-labeling method. Usually, astrocytes connect capillaries and neurons through perivascular pods and protrusions. They are an important part of the BBB and play a role in transporting nutrients and eliminating metabolites to neurons. Therefore, we speculate that overexpression of IFITM3 will change the morphology and function of astrocytes, destroy the structure of the BBB again, increase the permeability, aggravate the pathological damage of the animal nervous system, and then cancel the effect of rhBNP on therapeutic effects of cognitive impairment in septic mice.

There are some differences between our findings and previous studies on IFITM3, which may be attributed to the existence of *IFITM3* gene polymorphisms in mammals, the different molecular mechanisms of host immune activation to different pathogen stimuli, and the differential expression of IFITM3 in the central nervous system and peripheral tissue. However, the mechanism of action of IFITM3 in immune activation-related central nervous system diseases still needs more in-depth research.

This study also has certain limitations. In our study, we did not dynamically measure and intervene in the changes of cGMP content in the brain tissue of septic mice, detect the effection on the changes of cGMP on rhBNP regulating IFITM3 expression in the brain tissue of septic mice, and evaluate the therapeutic effect of rhBNP on cognitive dysfunction in animals, confirming the connection between rhBNP, cGMP, and IFITM3, revealing that the concentration change of intracellular second messenger cGMP is the key target of rhBNP therapeutic effect. The most upstream IFN signaling pathway can regulate IFITM3, which is closely related to cognitive dysfunction, clarify the key molecular mechanism of sepsis-related cognitive dysfunction, provide new opportunities for the application of rhBNP in the clinical treatment of SAE, and establish a solid theoretical foundation.

## 5. Conclusion

The activation of IFITM3 may be a potential new target for treating sepsis-associated encephalopathy (SAE), and it may be one of the key anti-apoptotic mechanisms in rhBNP exerting its therapeutic effect, providing new insight into the clinical treatment of SAE patients.

## Data availability statement

The original contributions presented in the study are included in the article/Supplementary material, further inquiries can be directed to the corresponding authors.

## Ethics statement

The animal study was reviewed and approved by the Ethics Committee of Xijing Hospital.

## Author contributions

NL, L-CH, and YG: conception and design of the research. NL, R-HM, D-YF, and JZ: acquisition of data. NL, E-FZ, and Y-NZ: analysis and interpretation of the data. R-HM and FG: statistical analysis. H-XJ and NL: obtaining financing and critical revision of the manuscript for intellectual content. NL, L-CH, and H-XJ: writing of the manuscript. All authors contributed to the article and approved the submitted version.

## Funding

This work was supported by Military Logistics Scientific Research Project (no. CLB20C036) and Natural Science Foundation of Liaoning Province (no. 2021-BS-030).

## Conflict of interest

The authors declare that the research was conducted in the absence of any commercial or financial relationships that could be construed as a potential conflict of interest.

## Publisher’s note

All claims expressed in this article are solely those of the authors and do not necessarily represent those of their affiliated organizations, or those of the publisher, the editors and the reviewers. Any product that may be evaluated in this article, or claim that may be made by its manufacturer, is not guaranteed or endorsed by the publisher.

## Figures and Tables

**Table 1:** 

ANP	Atrial natriuretic peptides
BBB	Blood–brain barrier
BNP	Brain-type natriuretic peptide
Caspase	Cysteiny aspartate specific proteinase
cAMP	Cyclic adenosine monophosphate
cGMP	Cyclic guanosine monophosphate
CLP	Cecal ligation and puncture
CNP	C-type natriuretic peptide
EPM	Elevated plus maze
ICU	Intensive care unit
IFITM3	Interferon-induced transmembrane protein 3
IFN	Interferon
MAC	Minimum alveolar concentration
MAP 2	Microtubule associated protein 2
NF-κB	Nuclear factor kappa B
NPR	Natriuretic peptides receptor
NPs	Natriuretic peptides
OFT	Open field test
rAAV	Recombinant adeno-associated virus
rhBNP	Recombinant human brain natriuretic peptide
SAE	Sepsis-associated encephalopathy
TNF-α	Tumor necrosis factor alpha
TLR4	Toll-like receptor
