# Corrigendum: Interferon-induced transmembrane protein 3 in the hippocampus: a potential novel target for the therapeutic effects of recombinant human brain natriuretic peptide on sepsis-associated encephalopathy

**DOI:** 10.3389/fnmol.2023.1294087

**Published:** 2023-09-27

**Authors:** Nan Li, Rui-Hang Ma, Er-Fei Zhang, Feng Ge, De-Yu Fang, Jing Zhang, Yan-Ning Zhang, Yan Gao, Li-Chao Hou, Hong-Xu Jin

**Affiliations:** ^1^Department of Emergency Medicine, General Hospital of Northern Theater Command, Shenyang, Liaoning, China; ^2^Department of Anesthesiology and Critical Care Medicine, Xijing Hospital, The Fourth Military Medical University, Xi'an, Shaanxi, China; ^3^Department of Anesthesiology, The Affiliated Hospital of Yan'an University, Yan'an, Shaanxi, China; ^4^Department of Chemistry, Liaoning University of Traditional Chinese Medicine, Shenyang, Liaoning, China; ^5^Department of Intensive Care Unit, Yue Bei People's Hospital, The Affiliated Hospital of Shantou University, Shaoguan, Guangdong, China; ^6^Department of Nephrology, General Hospital of Northern Theater Command, Shenyang, China; ^7^Department of Anesthesiology, Xiang'an Hospital of Xiamen University, Xiamen, Fujian, China

**Keywords:** sepsis-associated encephalopathy (SAE), recombinant human brain natriuretic peptide (rhBNP), interferon-induced transmembrane protein 3 (IFITM3), inflammation, apoptosis

In the published article, there was an error in the order of the corresponding authors. Instead of “Li-Chao Hou, houlichaohlc6@126.com; Hong-Xu Jin, jinhongxunjhx8@126.com”, it should be in the correct order of “Hong-Xu Jin, jinhongxunjhx8@126.com; Li-Chao Hou, houlichaohlc6@126.com”. We should also state Hong-Xu Jin is responsible for the integrity of the work as a whole. Li-Chao Hou contributes equally to this study as common corresponding author.

The authors apologize for this error and state that this does not change the scientific conclusions of the article in any way. The original article has been updated.

