# Supplementary material for: Interferon-induced transmembrane protein 3 in the hippocampus: a potential novel target for the therapeutic effects of recombinant human brain natriuretic peptide on sepsis-associated encephalopathy
Source: Front Mol Neurosci. 2023 Aug 1;16:1182005. doi: 10.3389/fnmol.2023.1182005 (PMC10436203; doi:10.3389/fnmol.2023.1182005)
Supplement: Supplementary file 1 [file Presentation_1.pdf]

Supplementary Materials

1. Cecal ligation and puncture-induced septic mouse model preparation

Our previous research methods established the sepsis mouse model by cecal ligation and puncture [4]. The animals fasted for 12 h before the operation, then were placed on a 37°C insulation board, inhaled 2% isoflurane (oxygen flow: 1 L/min) to induce anesthesia, their limbs were fixed, and the concentration of inhaled isoflurane was maintained at 1.2%–1.4% (oxygen flow rate, 1 L/min, i.e., one MAC for mice). The median abdominal incision was approximately 1.5–2 cm. The cecum was exposed, avoiding damaging mesenteric vessels and causing massive bleeding. The site about 50% from the end of the cecum was ligated with 5-0 silk thread. A 22 G needle was used to puncture the middle position of the ligated distal cecum once to ensure that the blood vessel was not penetrated. A small amount of feces in the intestine was squeezed out to ensure that the pinholes on both sides were not blocked. The amount of feces squeezed by each animal was ensured to be roughly the same, which can improve the stability of the model. The mice in the sham operation group were also subjected to the same open operation, and the cecum was also subjected to the same operation, except for ligation and puncture. The operation area was disinfected by smearing it with anerdian, 0.1 ml of 4% ropivacaine was injected into the incision, and lidocaine ointment was applied. The animals in each group were subcutaneously injected with normal saline (5 ml/100 g). An ertapenem powder injection (75 mg/kg) was infused intraperitoneally 6 h after the operation once a day for three consecutive days. The operation time of each animal was controlled within 10–15 min. Postoperative observation was carried out while the animals were placed in a 37°C incubator. After fully waking up, the animals were put back into the feeding

cage. Lidocaine ointment was applied daily for abdominal incision analgesia for three days.

2. Behavioral testing

Open field test and elevated plus maze

Open field test: The mice adapted to the laboratory environment one day before the test. The experimental open field platform is a 40 cm × 40 cm opaque box, artificially divided into 25 small squares of equal size. At the beginning of the test, the mice were placed in the central area and were free to move in the open field for 10 min. The video was collected, and the average movement speed in the last 5 min, the times crossing the squares, and the movement time in the central area were analyzed. Because the odor left by the animal in the open field has a major impact on the operation of the next animal, after each animal experiment, it was necessary to clean the experimental platform and target box with 70% alcohol to eliminate the guiding effect of the odor on the next animal.

Elevated plus maze test: After completing the open field test, the elevated plus maze test was carried out. The elevated plus maze test area: the cross-shaped medical plastic plate was placed at a height of about 1 m; the length, width, and height of each arm were 50 cm × 10 cm × 40 cm. The two transverse arms were closed, and the two longitudinal arms were open; a SONy # Super # HAD # CCD camera was installed about 1 m above to record the animals' track. The experimental animals were placed in the central area facing the open arm, and the action track of the animals was recorded for 5 min. The movement route of the mice and the time in the center were analyzed. After each animal was tested, the open field was cleaned and wiped repeatedly with 70% alcohol to remove the residual smell.
